# Diagnosis and Surgical Treatment of Renal Hydatid Disease: a Retrospective Analysis of 30 Cases

**DOI:** 10.1371/journal.pone.0096602

**Published:** 2014-05-05

**Authors:** Mulati Rexiati, Abudurezhake Mutalifu, Baihetiya Azhati, Wenguang Wang, Honglin Yang, Ilyar Sheyhedin, Yujie Wang

**Affiliations:** 1 Department of Urology, First Affiliated Hospital of Xin Jiang Medical University, Urumqi, Xin Jiang, China; 2 Yili Friendship Hospital, Yi Ning, Xin Jiang,China; 3 Department of Thoracic Surgery, First Affiliated Hospital of Xinjiang Medical University, Urumqi, Xinjiang, China; Fondazione IRCCS Ospedale Maggiore Policlinico & Fondazione D'Amico per la Ricerca sulle Malattie Renali, Italy

## Abstract

Echinococcosis (CE) is an infection which is caused by the larval stage of a tapeworm and is endemic in stockbreeding regions of developing countries. The kidney is the most commonly affected organ in the urinary tract. However, reports on renal hydatid disease are limited in the literature, and usually there are no specific clinical characteristics and promising operative methods. The purpose of this study is to assess the most appropriate surgical technique for the patient with urinary tract CE. We retrospectively analyzed thirty patients with renal hydatid cysts who received different surgical treatments in the urology department of the First Affiliated Hospital of Xinjiang Medical University from February 1985 to April 2010. Twenty patients were males and ten were females. The diagnostic accuracy was 74%, 87.5%, and 66.6% respectively by using of ultrasound, CT, and laboratory tests. Thirty patients were followed up for 1–15 years after surgery. One patient experienced a recurrence of renal CE. The ultrasound, CT, and immunological tests are an important means of diagnosis. The surgical treatment principle of renal hydatid should be based on residual renal function, hydatid cyst size, number, location, and surgical techniques to determine the surgical plan to retain the renal function.

## Introduction

Alveolar echinococcosis (AE) is a rare but life-threatening disease in humans. Hydatid cyst of the kidney is a very rare condition caused by the larval stage of Echinococcus granulosus. It is endemic in parts of the Middle East, South America, Australia, New Zealand, Alaska and stockbreeding regions of North-west China [1]–[3]. Dog is the definitive hosts of Echinococcus granulosus.Ship is the usual intermediate host, but humans are accidental intermediate hosts. In the human duodenum, the parasitic embryo penetrates the mucosa, allowing access to the blood stream, and enters the liver and lungs. The cysts are located in liver 75% times, lungs 15%, other organs 10%, isolated involvement of kidneys is rare and forms 1–5% of all hydatid disease in humans [4]–[7].The only definitive diagnostic sign of urinary tract CE is the presence of daughter vesicles in the urine, but this only occurs in 10–20% of patients with CE [8]. The common symptoms of renal CE are generally non-specific and subtle. Generally, most of the patients with renal hydatid may present atypical clinical manifestations, imaging features and acute renal colic and hydatiduria are common complications [9].

The treatment of renal hydatid cysts is surgery. Because of the lack of an absolutely effective systemic scolicidal agent surgical treatment offers the only hope of cure. The management of simple cysts is entirely for its symptoms or complications. Percutaneous treatment is alucrative, minimally invasive treatment option for management of symptomatic RC; however, there is wide variability in reporting of success depending on cyst size, sclerosant used definition of success, symptomatic improvement, and length of follow-up [10]–[11]. In general, results of simple aspiration are associated with very high recurrence rates (up to 90%) [12]–[13]. Single-incision laparoscopic surgery (SILS) was reported back in 1998 for cholecystectomy and appendicectomy [14]–[15], however, it did not gain momentum because of technical difficulties in steering standard laparoscopic instruments. Total or partial nephrectomy is recommended when the hydatid cyst lesions breaking into the collection system, rupture, infection and a serious kidney injury [16]–[18]. Nephrectomy and partial nephrectomy may result in loss of kidney function. In the present study, we retrospectively evaluate the most appropriate surgical technique for the patient who resented with renal and urinary tract CE.

## Materials and Methods

### Patients

The records of 30 consecutive patients with urinary tract CE who were hospitalized at the First Affiliated Hospital of Xinjiang edical University (Urumqi, Xinjiang) from February 1985 to April 2010. Data was obtained from hospital records. The Clinical features (symptoms, signs, location of lesions, serology, imaging, treatment) of all patients were analyzed. All patients underwent routine preoperative examinations, and results in all cases indicated general anesthesia and surgery would be tolerable (This preoperative examination included routine blood and urine tests, liver and renal function tests, measurement of electrolytes, and the coagulation function test. This retrospective study was approved by the Institutional Review Board of First Affiliated Hospital of Xin Jiang Medical University and written consent was obtained and written consent was given from the next of kin, caretakers, or guardians on the behalf of the minors/children participants, for their information to be stored in the hospital database and used for research.

### Diagnostic and Classification Criteria for Urinary CE

Urinary tract CE has similar structural features to the more common hepatic hydatid disease and can be characterized as caused by CL, CE1, CE2, CE3a, CE3b, CE4, or CE5 types, based on WHO/IWGE guidelines [5], [19]–[22]. All patients underwent ultrasound or computed tomography (CT) scanning. Patients with suspected echinococcosis underwent serological examination to determine the presence of antibodies to serum echinococcus granulosus cyst fluid antigen B (EgB), and alveolar echinococcosis-specific antigen (Em2).

### Surgical treatment

All patients were treated with various conservative or radical surgeries.Conservative surgery included simple internal endocyst excision plus drainage, internal capsule excision plus, and external pericyst wall resection [20]–[23]. Radical surgery included pericystectomy for the renal hydatid cyst and partial nephrectomy [21], [24], [25]. No patients were treated with laparoscopic surgery or PAIR (puncture, aspiration, injection, and reaspiration).

### Statistics

Continuous variables are expressed as medians and inter quartile range (IQR), depending on the distribution. The preoperative diagnostic accuracy of 3 methods was compared by Cochran's Q test. All statistical analyses were performed with SAS software version 9.2 (SAS Institute Inc., Cary, NC), and two-tailed p-value less than 0.05 was considered statistically significant.

## Results

Table 1 summarizes the preoperative patient demographic and clinical characteristics of 30 consecutive patients with urinary tract hydatid disease who were admitted to our department from February 1985 to April 2010. There were 20 males (66.7%) and 10 females (33.3%), and the median (IQR) age was 33 (5–66) years. 14 patients were Han ethnicity (46.7%), 8 patients were Kazak ethnicity (26.7%), 7 patients were Uyghur (23.3%),and other ethnicity make up 3.3% of the population.14 patients was cadres, teacher,students(46.7%),and 53.3% of the patients were Farmer.11 patients (36.7%) reported a history of contact with dogs or sheep. The most common symptom was lower back pain 12 (40.0%), upper abdominal pain 6 (20.0), 8 cases (26.7%) were found by physical examination, and 4 subjects (10.5%) reported non-specific symptoms. None of the patients experienced anaphylactic shock in response to treatment.

**Table 1:** Preoperative patient demographic and clinical characteristics (n = 30) with urinary tract cystic echinococcosis.

Age, median (IQR)	33 (5–66)
Gender, n (%)	
Male	20 (66.7)
Female	10 (33.3)
Ethnic group, n (%)	
Han	14 (46.7)
Kazakh	8 (26.7)
Uighur	7 (23.3)
Kirgiz	1 (3.3)
Occupation, n (%)	
Civil servant*	14(46.7)
Farmer	11(36.7)
Others▾	5 (16.7)
Contact with dogs or sheep, n (%)	
No	19 (63.3)
Yes	11 (36.7)
Surgery history, n (%)	
No	23 (76.6)
Liver hydatid disease	5 (16.6)
Renal Hydatid Disease	1 (3.3)
Kidney stone	1 (3.3)
Clinical symptoms, n (%)	
Lower back pain	12 (40.0)
Upper abdominal pain	6 (20.0)
Found by physical examination	8 (26.7)
Other 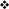	4 (13.3)

* 8 Cadres, 3 worker, 2 tteachers, and 1 nurse;

▾ 2 Students, 2 other workers and 1 child;

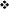
1Patients had recurrence of hydatid, 2 patients has non-specific symptom, 1 patients has fever.

Table 2 shows the clinical findings after surgery and follow-up. Lesions in the 8cases (26.6%) were located in the right kidney and 22 cases (73.3%) in the left kidney. The hydrated disease find only in kidney was 18 patients (60.0%), Kidney and liver was 6 (20.0%), Kidney, liver, and abdomen was 2 cases (6.6%), Kidney, liver and lung was 2 cases (6.6%), Kidney, pelvic, and hip was 1 cases (3.3%), and Kidney, retroperitoneal space was 1 cases (3.3%). Among the patient, 15 patients were underwent serological analysis.Serological test showed that 11 patients (73.3%) were positive for the EgB antigen and 8 cases (53.3) were positive for the Em2 antigen. The diagnosis was confirmed according to the pathological examination, 18 patients (60.0%) were classified as having E. granulosus renal disease only, and 12 patients (40.0%) with E. granulosus renal disease combined with hydatid disease in another organ(s). Seven patients (36.8%) suffered from a complication of increasing drainage fluid, and the drainage tube was delayed removed, none of the patients reported of leakage of urine. The median (IQR) duration of follow-up was 63 (14–177) months. At the last follow-up visit, 1 patient had evidence of renal CE recurrence (3.3%), although 3 patients (10.0%) with combined CE of other organs had non-renal recurrence of hydatid disease. Among the recurrence patients, 1 case was performed internal capsule excision.

**Table 2:** Introduction of clinical findings after surgery and follow-up (n = 30).

Location, n (%)	
Left kidney	22 (73.3)
Right kidney	8 (26.6)
Co-occurrence of other organs, n (%)	
Kidney only	18(60.0)
Kidney and liver	6(20.0)
Kidney, liver, and abdominal	2(6.6)
Kidney and retroperitoneal	1(3.3)
Kidney, liver and lung	2(6.6.2)
Kidney, plvic, and hip	1(3.3)
Serology▾, n (%)	
EgB	11 (73.3)
Em	8 (53.3)
Not examined	15 (0.0)
Disease type★, n (%)	
Granulosus renal disease	12(70.6)
Granulosus renal disease + hydatid disease in other organs	1 (5.8)
Other 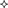	4(23.5)
Complication, n (%)	
Delayed drainage removing	3 (10.0)
Non	27(90.0)
Follow-up duration, median (IQR)	63 (14–177)
Renal recurrence of hydatid disease, n (%)	
No	29 (96.6)
Yes	1 (3.3)
Non-renal recurrence of hydatid disease, n (%)	
No	27 (90.0)
Yes	3 (10.0)

▾ Only 15 out of 30 patients underwent serological examination.

★ Pathologically confirmed after operation. Four patients had extrarenal ecurrence of hydatid disease.

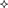
 4patents pathological findings was chronic inflammation.

**Figure 1:**
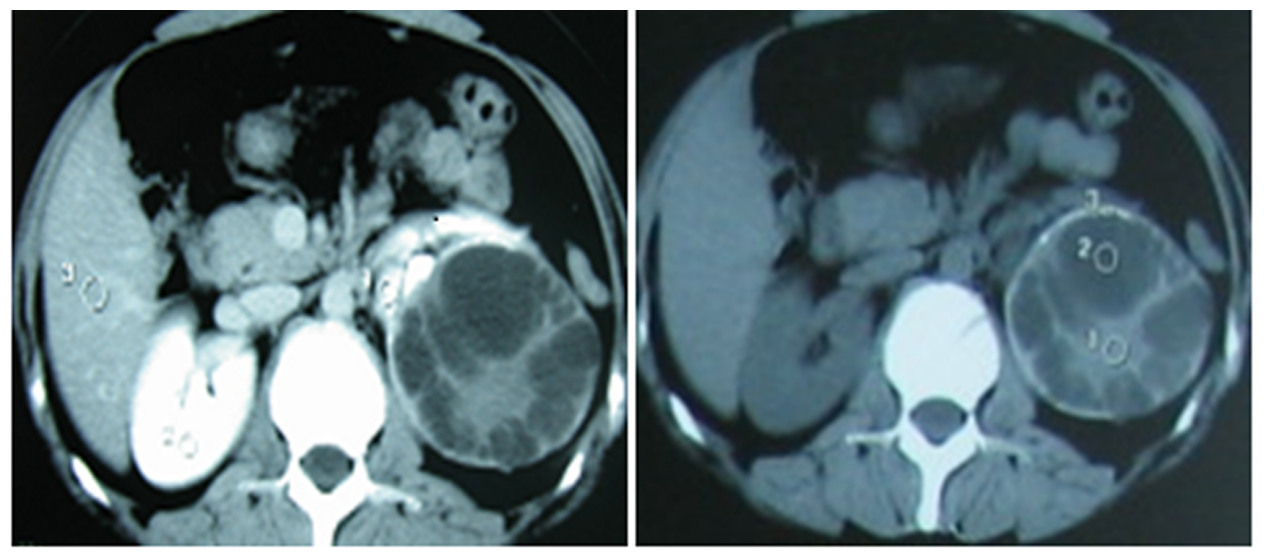
Type CE2 disease in a 28-year-old Uighur female patient. The patients has been found with an asymptomatic cystic mass in her left kidney by ultrasonography incidentally and diagnosed as renal hydatid cyst by further CT scanning one year before her admission to the hospital. She had a history of exposure to sheep and goats. And no family history of hydatid disease was identified. Physical examination observed a palpable mass in the left lumber region. Ultrasound revealed a univesicular cyst of 104×78×83 mm on the upper pole of the left kidney; CT confirmed the presence of hydatid cysts in the kidney.

**Figure 2:**
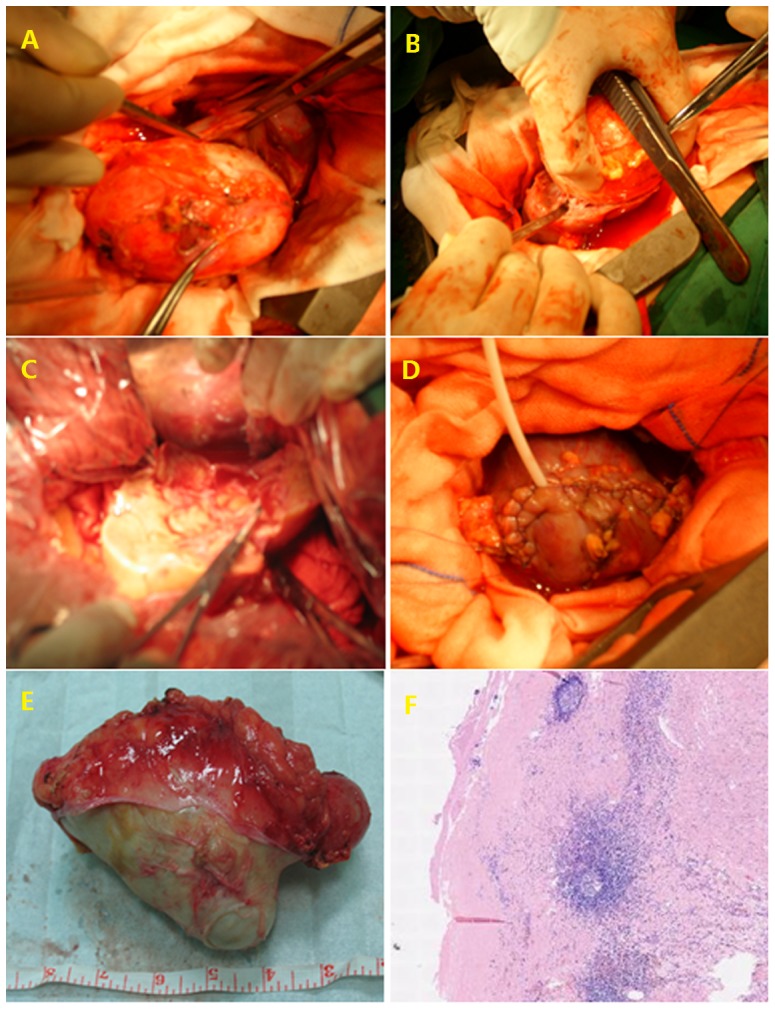
Intraoperative view of total external capsule excision. View of the hydatid cyst located in the in the left kidney (A); carefully dividing the intact ectocyst through the intra-adventitial space (B); Total cystectomy was performed A wound after complete removal of hydatid (A–B). Communicated with perirenal space during the procedure, and was closed by absorbable sutures and a F14 nephrostomy tube was placed in the calyx. A drainage was placed on perirenal space confirming no significant hemorrhage on surgical field, the wound was closed(C–D). Gross appearance and histopathologic examination of the cystic mass confirmed the hydatid disease, pathological analysis of the resected specimen was positive for scolices of Echinococcus granulosus (E–F).

Table 3 generalizes the classification of the imaging features according to the 2001 WHO/IWG-E classification of CE staging. On the basis of these guidelines, 2 patients (7.4%) had type CL, 5 patients (16.6%) had type CE1, 9 patients (33.3%) had type CE2, 2 patients (7.4%) had type CE3a, 4 patients (14.8%) had type CE3b, 3 patients (11.1%) had type CE4, and 2 patients (7.4%) had type CE5. All 9 patients with multivesicular hydatid cysts (CE2) had CE-specific signs (Figures 1 and 2). The CT images of all patients had only pericystic wall enhancement and no intracapsular enhancement. All of 30 patients underwent surgery, and postoperative confirmed diagnosis of renal CE disease was determined by pathological examination. 3 patients receiving simple internal capsule excision suffered postoperative increasing drainage fluid, and the drainage tube was removed within 2 months, and none of the patients reported of leakage of urine.

**Table 3:** Classification of imaging results based on the WHO/IWG-E classification of cystic echinococcosis (n = 27).

WHO/IWG-E classification	Image characteristics	No (%)
CL	Univesicular, cystic lesion with uniform echoes, clear boundary, thin visible wall. If it is a hydatid cyst, it is active.	2(7.4)
CE1	Univesicular anechoic cyst. Presence of hydatid sand, snow flake sign and double wall sign. The hydatid is active	5(16.6)
CE2	Multivesicular, multiseptated cysts; cysts septations produce “wheel-like”structures, and presence of daughter cysts is indicated by “rosette-like” or “honeycomb-like” structures.	9(33.3)
CE3a	Detachment of laminated membrane from the cyst wall visible as “big snake sign” or as “water-lily sign”. The hydatid status is transitional.	2(7.4)
CE3b	Intracystic shadow of the daughter vesicles and solid septation, manifested as complex cyst shadow. The hydatid is dying.	4(14.8)
CE4	Heterogenous hypoechoic or hyperechoic contents ecurrence of hydatid disease.	3(11.1)
CE5	Intracystic solid degeneration and calcification of the cystic wall. The hydatid is inactive	2(7.4)

Table 4 summarizes the type of operations performed in 30 CE patients. Internal capsule excision was performed in 23 cases, 5 cases received external capsule excision capsule excision, 1 patient had partial nephrectomy, and the 1 patient underwent nephrectomy.

**Table 4:** Types of surgery, presence of complications, and recurrence of non-renal hydatid disease (n = 30).

Type of Surgery	Total No	Complication (%)	Non-renal recurrence (%)
Internal capsule excision	23	3(13.0)	3 (13.0)
External capsule excision	5	0(0.0)	0(0.0)
Partial nephrectomy	1	0(0.0)	0(0.0)
Total nephrectomy	1	0(0.0)	0(0.0)

Table 5 illustrates the preoperative diagnostic accuracy of hydatid disease based on different methods. The preoperative diagnostic accuracy rate was 74% for ultrasound, 87.5% for CT, and73.3% for serology when final pathological examination was used as the gold standard for diagnosis. These differences were not significant (p = 0.223). Correct diagnosis was achieved in 20 of 30 patients before surgery, and the remaining 10 patients were diagnosed during surgery or by postoperative pathological results.9 of the 30 patients had typical imaging feature of hydatid disease stage CE2 and it was confirmed during or after surgery. Six patients had previous histories of surgery due to CE, which provided diagnostic clues. One patient who had undergone ipsilateral kidney stone surgery experienced. Twelve patients have a chief complain of lower back pain and underwent further ultrasound or CT examination; one of these cases misdiagnosed as renal tumor, and one patient with lower back pain underwent CT scans and were initially misdiagnosed as having suspected kidney stone.

**Table 5:** Pre-operative diagnostic accuracy of hydatid diseasebased on different methods (n = 30).

Diagnostic Method	Total No	Correctlydiagnosed cases No (%)	P
Internal capsule excision	23	3(13.0)	3 (13.0)
External capsule excision	5	0(0.0)	0(0.0)
Partial nephrectomy	1	0(0.0)	0(0.0)
Total nephrectomy	1	0(0.0)	0(0.0)

Cochran's Q test was used to compare the pre-operative diagnostic accuracy rate of ultrasound, computed tomography, and serology.

## Discussion

In endemic countries, renal hydatid disease is a rare and challenging condition to diagnose. Imaging plays the key role in diagnosing and staging of CE, whereas, there are no specific signs or symptoms for renal hydatid disease usually remains asymptomatic for years and serology has only a minor, confirmatory role due to high rates of false negative results [26]–[27]. Presenting symptoms of cystic echinococcosis are highly variable and the most common symptoms are palpable mass, flank pain, hematuria, malaise, fever and hydrator [28]. In our study, 12 of 30 patients with renal or urinary tract CE showed lower back pain, 6 cases has a common symptoms of upper abdominal pain, 8 asymptomatic cases were found by physical examination, 2 patients has fever, 1patient reported recurrence of hydatid, 1 subject exhibited loss of appetite, none of the patients experienced hematuria and hydrator,and the preoperative clinical diagnostic accuracy was 66.6%. Lower back pain is the most common symptoms of urinary tract CE.However; a non-specific symptoms result does not exclude or confirm the diagnosis of renal hydatid disease, and urinary tract CE is often misdiagnosis which brings about major health consequences. In our research, 8 asymptomatic cases ware found his urinary tract CE by their physical examination,so,it is advisable that the regular medical check-ups is beneficial for commonwealth, especially the people living in the endemic countries.in our study, left kidney CE was 73.3% that higher than right kidney. Most of the report about renal hydatids are the one case report because of the low incidence. So it is hard to conclude which side of kidney has high morbidity or whether have a statistical differences, though there are some reports. Imani F [29] reported 10 patients with the renal hydatids, involving the left kidney in 8 cases and the right kidney in 2 cases. Göğüş C [30] et al report 20 patients about renal hadatids, 14 cases on the left side and on the right side in 6. In the another study about kidney hydatids include 18 patients, 12 on left kidney, 6 on the right [31]. Most hydatids occur in the right lobe of the liver, because of anatomical structure of the liver vein. May be it is same to the kidney hydatids that left renal artery shorter than right renal artery, so there are high chance to Hydatid larvae spread to left kidney first.

Imaging plays the key role in diagnosing and staging of CE, whereas serology has only a minor, confirmatory role due to high rates of false negative results [32]. Through ultrasound is the most essential tool for hydatid disease and clearly demonstrates the floating membranes, daughter cysts, and hydatid sand characteristically seen in purely cystic lesions and the bases for the international classification of ultrasound images of cystic echino-coccosis produced by WHO expert group [33]–[34]. Echocardiography is the preferred diagnostic method because of its low cost and availability.However, it is sometimes inadequate in making the initial diagnosis [35]. CT is superior to other imaging modalities in observing intracystic gas, minute calcifications, and in anatomical mapping, Cysts may be identified as single or multiple, and uni-or multilocular [36].Moreover, serological observation of echinococcosis appear to be a comprehensive and useful tool to monitor changes of transmission dynamics in humans and provide ‘warning signals’ to decision makers for the instigation of specific control measures against the disease [37].In this research, 27 of 30 patients were examined by ultrasound, the preoperative diagnostic accuracy rate was 74% for ultrasound. The WHO/IWG-E classification system for CE diagnosis and treatment is based on ultrasound medical imagery and classifies hydatid cysts as stage CL, CE1, CE2, CE3a, CE3b, CE4, or CE5 (Table 3). WHO-IWGE developed a standardised classification that could be applied in all settings to replace the plethora of previous classifications and allow a natural grouping of the cysts into three relevant groups: active (CE1 and 2), transitional (CE3) and inactive (CE4 and 5) (WHO and Echinococcosis, 2003). Generally, active cysts are need to intervention including chemical therapy or surgery,because active cysts cause the lesions, spread to the other organs. inactive cysts are don't need to intervention, they lost viability,So they can go to only expectation. Among them 2 patients had type CL, 5 patients had type CE1, 9 patients had type CE2, 2 patients had type CE3a, 4 patients had type CE3b, 3 patients had type CE4, and 2 patients had type CE5. Type CE1 and CE2 cysts are considered as a active and fertile with viable protoscoleces; CE3a and CE3b cysts are in a transitional stage when the integrity of the cyst compromised; and CE4 and CE5 cysts are inactive and degenerative [20].in our study, CE1, CE2,and CE3 type of CE More than other types. Serological test was performed in 15 urinary tract CE by using ELISA method [38]. Serological test was performed in 15 of 30 urinary tract CE patients, preoperative serological analysis of EgB and Em2 antibodies using the Rapid Diagnostic Kit for Human Echinococcosis.That preoperative diagnostic accuracy rate of serological analysis was (73.3%) lower than that of ultrasound (74%) or CT scans (87.5%), although these differences were not significant. In general, imaging by CT or ultrasound is considered the main tools for diagnosis, and serology and other tests are considered complementary [26]. Our results indicate that CT had a higher diagnostic accuracy rate than ultrasound and serological examination; the result was consistent with the literature.

Kidney-sparing surgery is performed whenever possible [39].Owing to the lack of an entirely powerful systemic scolicidal agent, surgical treatment offers the only hope of recovery. The procedure of preference is the simple excision of the cyst. When the kidney is damaged, nephrectomyis necessary. Medical management of renal hydatidosisis far from being are realistic alternative to surgery and should be considered as adjuvant therapy [40]–[42].Chemotherapy,as an adjuvant therapy,with or without puncture aspiration-injection-re-aspiration (PAIR) is suitable for inoperable renal hydatid disease [43]–[44], however,none of patients in this study were receive methodology. Surgery may cure the patient completely but does not totally prevent recurrence. Generally, use of albendazole six month after internal capsule excision for prevention. After Pericystectomy don't need to use albendazole. And other site abendazole are indicated for inoperable patients with liver or lung CE, patients with multiple cysts in two or more organs, or peritoneal cysts [45].Using alberndazole for one week to one month before surgery may reduce the intraoperative tension of the CE cyst, prevent CE spread during puncture, and may kill or reduce the activity of Echinococcus larvae. Continuous use of albendazole for 3 months after surgery may also reduce postoperative recurrence, especially when cystic fluid has spread during surgery [46]. A recent paper comparing different perioperative ABZ regimens concluded that ABZ is an effective adjuvant therapy in surgical treatment of liver CE [47]. ABZ has been proven teratogenic in rats and rabbits. Physiological exposure to ABZ and its principal metabolite, ABZ sulfoxide, in early human pregnancy is substantially lower (perhaps. 10–100 times) than in the animal species in which teratogenic or embryotoxic effects have been recorded. Therefore, the risk of fetal exposure from the recommended therapeutic dose is probably very small. Despite the fact that no abnormal birth outcome has been observed following ABZ administration during pregnancy, treatment of gravid or potentially gravid females should be avoided, unless the benefit of treatment significantly outweighs the potential risk to the developing fetus [48]. Treatment interruptions were felt to be required because of the limited long-term toxicity data available in the early days of use [45].

All of 30 patients received surgery, Internal capsule excision was performed in 23 cases, 5 cases undergone external capsule excision capsule excision (See Fig 2), 1 patient had partial nephrectomy, and the 1 patient underwent nephrectomy.3 patients treated with internal capsule excision suffered postoperative increasing drainage fluid, and the drainage tube was removed within 2 months, and none of the patients reported of leakage of urine. 1 patient underwent internal capsule excision had renal CE recurrence, and although 3 patients had non-renal recurrence of hydatid disease.

According to our study, it is suggest that external capsule excision for the treatment of renal hydatid cysts with considerable size and no communication with the collecting system could be a safe, effective management with lower morbidity and local recurrence rates. It will be a useful supplement in the treatment of renal and other organ hydatid disease. The limitation is that the study design is insufficient for us to reach conclusions due to lack of results from the large numbers and long-term follow-up studies. Laparoscopic surgery is not first choice for the operation. Laparoscopic surgery is a technical option in selected cases but has the high risk of complications including spillage, secondary Hydatids, Postoperative urine leakage. Any effort made to avoid fluid spillage is recommended, including protection of peritoneal tissues. In the future we will perform the Laparoscopic surgery for the cyst which far from collecting system, small and no adhesion with adjacent organs.

The surgical treatment principle of renal hydatid should be based on residual renal function, hydatid cyst size, number, location, and surgical techniques to determine the surgical plan; it should be possible to select the complete removal of lesions, relapse prevention, and to retain the renal surgery.
